# Exploring the Transcriptional Responses of Wheat to Sowing Density and Cultivar Mixtures

**DOI:** 10.1002/pei3.70203

**Published:** 2026-10-30

**Authors:** Laura Mathieu, Amandine Chloup, Louis Gicquelet, Justin Savajols, Pierre Roumet, Jean‐Benoît Morel, Martin Ecarnot, Elsa Ballini

**Affiliations:** ^1^ PHIM Plant Health Institute, Univ Montpellier, INRAE, CIRAD, Institut Agro, IRD Montpellier France; ^2^ PHIM Plant Health Institute, Univ Montpellier, CIRAD, Institut Agro, INRAE, IRD Montpellier France; ^3^ UMR AGAP Institut, Univ Montpellier, CIRAD, INRAE, Institut Agro Montpellier France

**Keywords:** cultivar mixtures, gene expression, mesocosm, plant–plant interactions, sowing density, wheat

## Abstract

Cultivar mixtures enhance diversity in fields, offering multiple agronomic benefits while remaining compatible with mechanized agriculture. However, mechanization introduces limitations, such as sowing density, which can influence crop yield. In order to assess the impact of agricultural practices on the plant physiological state, targeted gene expression analysis provides a useful approach. The molecular responses of crops to sowing density and varietal mixtures, particularly in wheat, remain poorly explored. In this study, we examined the transcriptional responses of two binary mixtures of wheat cultivars grown at two sowing densities over two years in semi‐controlled mesocosm conditions, using RT‐qPCR. Despite notable year‐to‐year variation in gene expression, consistent transcriptional trends were identified. Sowing density significantly influenced gene expression, particularly in pathways related to carbon metabolism, with cultivar‐specific effects. Interactions between sowing density and cultivar mixtures were also observed, with different gene expression patterns across wheat cultivars. While the direct influence of cultivar mixtures on gene expression was limited, three specific genes—particularly in durum wheat—were differentially regulated under mixture conditions. These findings indicate that external environmental factors play a dominant role in shaping gene expression, but the molecular effects of sowing density and varietal diversity should be further considered to optimize sustainable agricultural practices.

## 1 Introduction

Wheat is a major global crop, dominating cereal production in France and ranking second worldwide (Intercéréales 2021). Bread wheat ( *Triticum aestivum* ) is the most widely cultivated species, while durum wheat (*Triticum turgidum var. durum*) plays a key role in hot and dry regions (Shewry and Hey 2015). Modern wheat production faces the dual challenge of increasing or maintaining yield while reducing environmental impacts. Agroecological approaches aim to address this challenge by promoting crop diversification and optimizing management practices to leverage ecosystem services (Ewert et al. 2023). Among these strategies, varietal mixtures increase within‐field genetic diversity and have been shown to stabilize yield and reduce disease severity, while remaining compatible with mechanized agriculture (Borg et al. 2018; Kristoffersen et al. 2019; Li et al. 2023). However, key management factors, notably sowing density, strongly influence crop performance and interact with diversification strategies, highlighting the need for optimized practices. Achieving this optimization requires not only agronomic and physiological knowledge but also a mechanistic understanding of how wheat integrates biotic and abiotic signals—such as resource availability and neighbor presence—at the molecular level to shape development and productivity.

Wheat yield responses to sowing density can be highly variable, making the definition of an optimal plant density a persistent challenge. While low densities reduce resource use efficiency and yield (Whaley et al. 2000), high densities can increase production costs and decrease yield through lodging and disease pressure (Lloveras et al. 2004; Laghari et al. 2011). Empirical studies report inconsistent yield responses to plant density, with negative (Geisler et al. 2012; Bastos et al. 2020; Wang et al. 2022), neutral (Dueri et al. 2022), or even positive effects (Calderini et al. 2021) depending on genotype and environment. At the physiological and molecular levels, high plant density alters key developmental and metabolic processes, including accelerated senescence, reduced photosynthesis, impaired root and shoot growth, and modified nutrient acquisition (Geisler et al. 2012; Masclaux et al. 2012; Dai et al. 2014; Muñoz‐Parra et al. 2017; Zheng et al. 2017; Postma et al. 2021; Beaugendre et al. 2022). These responses are associated with density‐dependent transcriptional reprogramming affecting hormone signaling, particularly auxin‐related pathways, as well as genes involved in defense, nutrient deficiency, light perception, photosynthesis, and stress responses (Masclaux et al. 2012; Muñoz‐Parra et al. 2017; Postma et al. 2021; Beaugendre et al. 2022). However, reported molecular responses are often contradictory across studies (Geisler et al. 2012; Dai et al. 2014; Zheng et al. 2017), highlighting the context dependency and limited characterization of density‐induced transcriptional regulation in wheat. This variability underscores the need for further molecular investigations to better characterize how sowing density affects wheat physiology and yield.

Varietal mixtures modify plant development, susceptibility, and productivity through plant–plant interactions whose underlying mechanisms remain poorly understood. In wheat, mixtures generally increase yield (Borg et al. 2018), which was attributed to differences in tillering dynamics (Gawinowski et al. 2022; Stefan et al. 2025). Studies across crop and model species show that neighbors influence both above‐ and belowground traits, including root architecture and nutrient acquisition, likely mediated by root exudates (Fang et al. 2013; Mathieu et al. 2024). At the molecular level, plant–plant interactions trigger transcriptional reprogramming affecting photosynthesis, defense responses, hormone signaling, nutrient transport, and developmental pathways (Subrahmaniam et al. 2018; Becker et al. 2023). In particular, upregulation of defense‐related genes is frequently observed in response to conspecific neighbors and is associated with changes in disease susceptibility in both intra‐ and interspecific mixtures (Biedrzycki et al. 2011; Xu et al. 2015; Chen et al. 2019; Pélissier et al. 2021; Mathieu et al. 2025). Competitive environments also induce gene expression reprogramming linked to accelerated development and reproductive allocation (Bonser 2013; Subrahmaniam et al. 2018). Despite these findings, molecular responses to neighbors—especially in major crops such as wheat—remain sparsely characterized, limiting our ability to predict how varieties will perform in mixtures.

The interaction between sowing density and varietal diversity has mainly been examined through architectural and yield‐related traits. At low densities, spatial separation limits plant competition, whereas increasing density intensifies overlap among neighboring plants and competition for resources (Bulson et al. 1997; Beaugendre et al. 2022). While biomass and productivity often increase with both density and diversity (He et al. 2005; Newberger et al. 2023), these effects are highly context‐dependent (Lortie et al. 2016). Notably, the diversity‐productivity relationship tends to weaken at high densities compared with low densities (He et al. 2005) and depends strongly on neighbor identity (Stefan et al. 2022). Density‐diversity interactions are also associated with changes in the soil microbiome (Newberger et al. 2023) and reduced weed pressure (Olsen et al. 2006). These interactions are likely to depend on cultivar identity, as modern cultivars have been bred for optimal performance under high‐density conditions. Maximizing the benefits of varietal mixtures may therefore require jointly optimizing sowing density and cultivar choice. Despite evidence that sowing density modulates the outcomes of cultivar mixtures, studies have so far focused exclusively on phenotypic responses. The molecular mechanisms underlying density‐mixture interactions remain unexplored, leaving open whether reduced signaling at low density or increased competition at high density alters plant responses to neighbors at the transcriptional level.

In this study, we evaluated whether molecular responses captured through molecular phenotyping can be reliably assessed under semi‐controlled conditions that bridge the gap between laboratory and field experiments. By moving beyond greenhouse pot experiments, this approach enables a more realistic assessment of density‐dependent molecular responses in wheat. Using a RT‐qPCR chip targeting 96 wheat genes involved in diverse biological processes, we investigated the effects of sowing density, cultivar mixture, and their interaction on wheat transcriptional responses. We hypothesized that (i) sowing density and cultivar mixtures significantly influence wheat molecular responses, and (ii) the effects of plant–plant interactions on gene expression depend on sowing density.

## 2 Materials and Methods

### 2.1 Biological Material

The study utilized two cultivated varieties of bread wheat (Venezio and Soissons) and two inbred lines (EPO 122 and EPO 145) of durum wheat from an Evolutionary Pre‐breeding pOpulation (EPO) developed at INRAE Montpellier (David et al. 2014). The lines were selected for their similar traits in aerial architecture in order to minimize aboveground competition and variation in canopy microclimate and to focus on differences arising from belowground interactions.

### 2.2 Experimental Design

Each wheat cultivar was grown alone (in “pure”) or in mixture with another cultivar of the same species (in “mix”). Each blend type (pure or mix) was sown at two densities: 192.5 plants/m^2^ (low density) and 400 plants/m^2^ (high density), and each condition (corresponding to the combination of mixture type and density) was replicated in two independent plots, which were randomly positioned in the experimental trial (Figure 1A). Each mesocosm covered an area of 0.8 m^2^, and seeds were sown in a regular grid to ensure uniform spacing between plants both within and between rows (Figure 1B). The experiment was replicated over two years. In each year, treatments were randomly reassigned to mesocosms to avoid any carry‐over or positional effects. Seeds were sown on 02/12/2023 for the 2023 experiment, and on 10/01/2024 for the 2024 one; these sowing dates were selected to match local agronomic practices under the experimental conditions.

**FIGURE 1:**
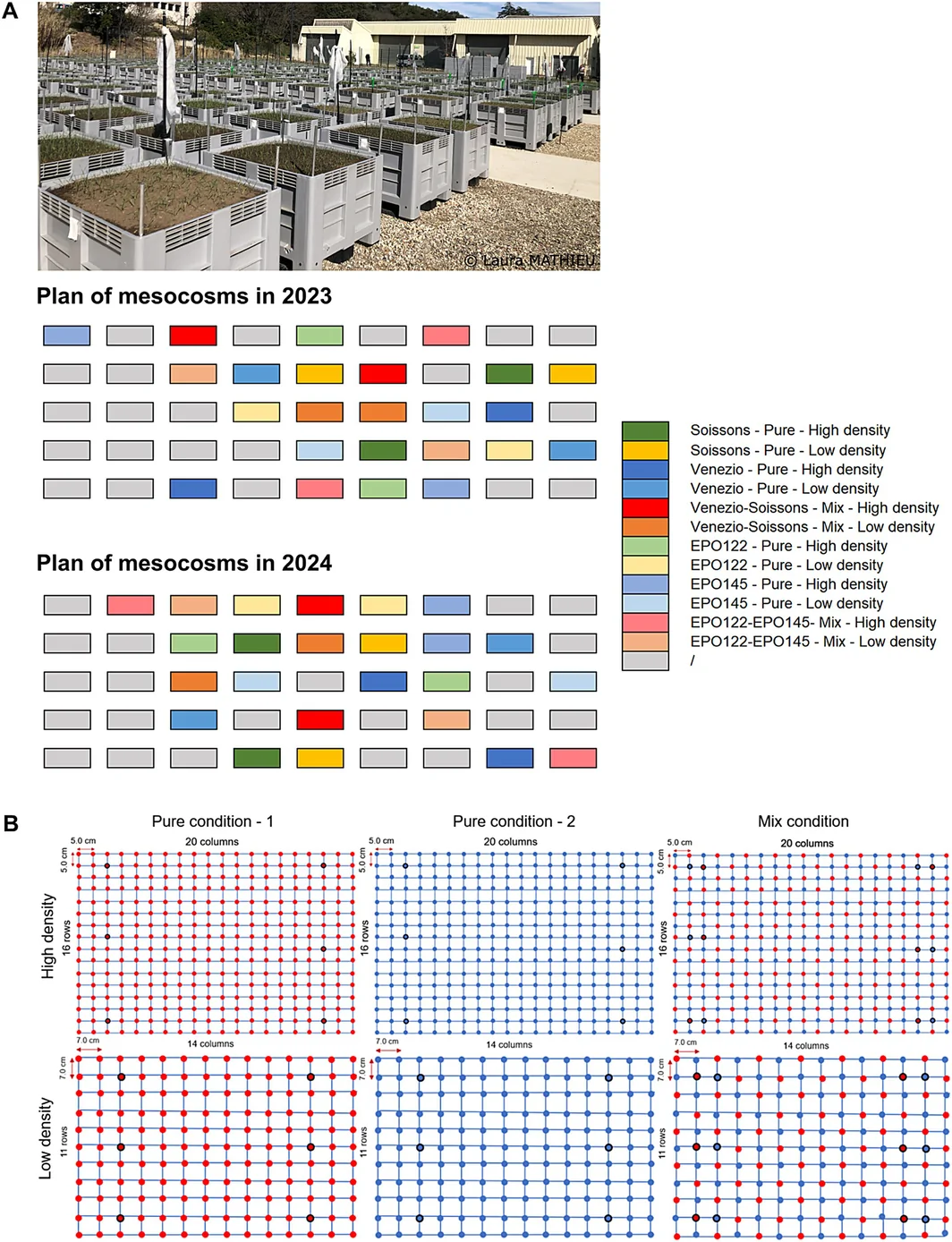
Experimental design to study the effects of sowing density and cultivar mixture under mesocosm conditions. (A) Photograph of the experimental site and scheme of the mesocosm layout for each year. (B) Diagram of the mesocosm sowing plan, with harvested plants circled in black.

### 2.3 Experimental Site

The study was conducted in 2023–2024 at the Agropolis Resource Centre for Crop Conservation, Adaptation and Diversity (ARCAD) mesocosm in Montpellier (43°38′ N, 3°51′ E). The climate is Mediterranean. Meteorological data (Figure S1) was obtained through an INRAE meteorological station located approximately 800 m away from the experimental site.

The soil was analyzed by Auréa, an analytical laboratory, and characterized as a silt. Different soil properties were measured before the experiment: $pH_{H_{2}O}$ = 7.9, pH_KCl_ = 7.2, total CaCO_3_ = 1.4%, cation‐exchange capacity (CEC) = 11.7 meq/100 g, P_2_O_5_ = 63 mg/kg, K_2_O = 251 mg/kg, organic matter = 4.7 g/kg, total carbon = 2.73 g/kg.

### 2.4 Sample Collection

Leaf samples were collected to analyze the gene expression at the 3rd expended leaf tissue stage. The samples were collected on 15/02/2023 for the 2023 experiment, and on 04/03/2024 for the 2024 one.

Each tube sampling consists of a pool of the 3rd expanded leaf tissue from 3 different plants of the same genotype collected in the same planter. For each genotype per planter, two tubes were collected (each comprising a pool of 3 different leaves). After collecting, the central part of the 3 leaves was quickly cut into 0.5 cm sections, put into a 1.5 mL Eppendorf tube, and immediately stored in liquid nitrogen. The sampled plants' localization (row and column coordinates) is identical for each planter (Figure 1B).

### 2.5 Primer Design for the High‐Throughput RT‐qPCR Chip

A systematic literature review was conducted to identify genes involved in selected processes (Figure S2, Table S1). If available, we preferably selected studies showing gene transcriptional regulation (by RT‐qPCR, microarray or RNA‐seq) in wheat young seedling leaf related to a targeted process. When we could not find studies reporting transcriptional regulation of genes under a targeted process, we selected studies showing the implication of genes using mutant approaches. If available, we selected already designed PCR primers based on our bibliography analysis. However, if no primers were already published for the gene targeted, we designed our own primers. The design of primers followed a set of stringent criteria, as generally suggested in RT‐qPCR protocols (e.g., PrimerExpress Software v2.0 Application Manual, Applied Biosystems). To minimize the risk of amplifying contaminating genomic DNA, primers spanning at least one exon‐exon junction, or annealing to different exons, were designed when possible. The specificity of each primer was confirmed by comparing its sequence with all predicted wheat coding sequences (CDS) using the Primer3Plus software to ensure that at least one primer of each pair targets a unique site within the set of predicted wheat CDS. Primers specificity and amplicon length were checked for each pair of primers using MFEprimer3.1 and verification of hairpin and homo/heterodimerization was checked with idtdna OligoAnalyzer.

### 2.6 RNA Extraction

Total RNA was extracted using the protocol described in Delteil et al. (2012). Initially, frozen leaf tissues were ground in liquid nitrogen. Cells were lysed by adding 1 mL of Tri‐reagent to the powdered sample. Nucleic acids were then separated by adding 200 μL of chloroform, incubating for 10 min at room temperature, followed by centrifugation at 13000 *g* for 15 min at 4°C. The upper aqueous phase was collected, and 560 μL of isopropanol was added to precipitate the nucleic acids overnight at −80°C, followed by centrifugation at 13000 *g* for 20 min. The resulting pellet was washed twice with 500 μL of 70% ethanol and centrifuged at 13000 *g* for 5 min at 4°C and then air‐dried for 10 min before resuspension in 50 μL of ultrapure water on ice for 1 h. DNase treatment was performed on 50 μL of RNA using the TermoFisher kit, followed by purification. 200 μL of RNA was mixed with 200 μL of phenol:chloroform:alcohol (25:24:1), and centrifuged at 16,000 *g* for 10 min at 4°C. The upper phase was collected and precipitated with 18 μL of 3 M sodium acetate and 495 μL of pure ethanol at −80°C for 1 h, followed by centrifugation at 16000 *g* for 30 min. The pellet was washed with 1 mL of 70% ethanol, centrifuged for 5 min, dried, and resuspended in 50 μL of ultrapure water. RNA quality and quantity were assessed using a Nanodrop spectrophotometer and verified for integrity via 2% agarose gel electrophoresis. The absence of DNA contamination was confirmed by qPCR using the Promega kit with EGM34 and EGM35 primers. For cDNA synthesis, RNA was diluted to 625 ng/μL in 8 μL, combined with 2 μL oligo(dT) and 3 μL H_2_O, and incubated at 95°C for 5 min. Subsequently, 4 μL 5× buffer, 2 μL 10 mM dNTPs, and 1 μL RT‐MMVL enzyme (Promega) were added, and the mixture was incubated at 37°C for 30 min to generate cDNA.

### 2.7 Fluidigm Chip Preparation

Gene expression analysis was conducted using a Fluidigm chip assay. Samples obtained post‐reverse transcription were pre‐amplified with Fluidigm products following the ‘Pre‐amplification of cDNA for Gene Expression with delta GeneAssays’ protocol provided by the manufacturer (Biomark, Stan‐dard Biotools Inc. San Francisco, CA, USA). Initially, 0.5 μL of 100 μM forward and reverse primers were diluted in 200 μL of DNA suspension buffer. A pre‐mix was prepared by combining 105.6 μL of PreAmp Master Mix, 52.8 μL of the diluted primer mix, and 237.6 μL of DNase‐free H_2_O. Each well of a 96‐well plate was filled with 3.8 μL of the pre‐mix and 1.75 μL of 1/10 diluted cDNA sample. The plate was incubated with the following program: 2 min at 95°C, followed by 10 cycles of 15 s at 95°C, and 4 min at 60°C. Post pre‐amplification, a clean‐up reaction was performed by preparing an exonuclease mix (168 μL of DNase‐free H_2_O, 24 μL of Exonuclease I Reaction Buffer, and 48 μL of Exonuclease I at 20 U/μL). Each well received 2 μL of this mix, and the plate was incubated for 30 min at 37°C for digestion, followed by 15 min at 80°C to inactivate the enzyme. Subsequently, 43 μL of DNA suspension buffer was added to each well, diluting the samples to one‐tenth of their concentration, and the plate was stored at −20°C. For Fluidigm chip preparation, the “primers” and “samples” plates were arranged. The “primers” plate was prepared by mixing 3 μL of assay loading reagent, 2.7 μL of DNA suspension buffer, and 0.3 μL of 100 μM primers (forward + reverse). The “samples” plate mix contained 360 μL of SoFast EvaGreen Super Mix and 36 μL of DNA binding dye sample loading reagent, with 3.4 μL of this mix and 2.75 μL of pre‐amplified samples added to each well, achieving a final volume of 5 μL per well. The software to obtain the data was Fluidigm Real‐Time PCR Analysis, with the quality threshold set at 0.65. Gene expression for 96 genes (Table S1), identified from the literature, was measured and normalized using the 2^ΔΔCt^ method (Livak and Schmittgen 2001), with 2 reference genes Tubulin and ATPase Ta54227, implemented using the R package FLUIDIGR. Genes with undetected expression (CT = 999) were excluded from the dataset. Any gene or sample with 20% or more missing data was removed from the analysis.

### 2.8 Data Analysis

All statistical analyses were performed in R v.4.3.1.

Transcriptional profiles were visualized using heatmaps generated with the gplots package and Principal Component Analysis (PCA) performed with the FactoMineR and factoextra packages. Statistical analysis focused on genes displaying consistent expression trends across both years of the experiment. For each gene, statistical analyses were then performed separately within each year. The effects of plant density (low vs. high density), blend type (pure vs. mix), and their interaction were evaluated using a non‐parametric ANOVA, followed by Benjamini−Hochberg correction (Equation 1). Boxplots were generated using the ggplot2, see, and stats packages to visualize significant differential gene expression. Genes were classified as differentially expressed if they showed significant changes in expression in at least one year, with a similar expression trend in the other year.

(1) $$y_{\mathrm{ijk}}=\mu +\alpha_{i}\;B_{i}+\beta_{j}\;D_{j}+\gamma_{\mathrm{ij}}\;{\mathrm{BD}}_{\mathrm{ij}}+\varepsilon_{\mathrm{ijk}}$$

where y_ijk_ is the phenotypic observation; μ is the intercept; α_i_, β_j_, and γ_ij_ correspond to effects for blend type i, density condition j and interaction between blend type i and density j, respectively; B_i_, D_j_, and BD_ij_ correspond to blend type i, density condition j and interaction between blend type i and density j, respectively; and ε_ijk_ is the error term.

## 3 Results

### 3.1 Wheat Transcriptional Profiles Were Affected by Year‐To‐Year Variation

To visualize the annual variation in transcriptional profiles across the wheat cultivars, we performed a principal component analysis (PCA). The results revealed clear separations by year for both bread wheat cultivars (Venezio, Soissons) and durum wheat cultivars (EPO122, EPO145) (Figure 2). Transcriptomes of bread wheat cultivars showed greater dispersion in 2023 compared to 2024, likely reflecting increased environmental heterogeneity or more pronounced stress conditions during the 2023 growing season (Figure 2). These observations highlight the significant impact of year‐specific environmental conditions on gene expression. To mitigate the effect of interannual variability, we focused our subsequent analysis on genes that demonstrated consistent expression patterns across both years (Table S2). This filtering process ensured that the analyses were robust and reflective of trends independently of annual variation. For the density effect analysis, we obtained 24 genes in Venezio, 27 in Soissons, 37 in EPO122, and 35 in EPO145 (Table S2). For the mixture effect, we analyzed 31 genes in Venezio, 24 in Soissons, 43 in EPO122, and 41 in EPO145 (Table S2).

**FIGURE 2:**
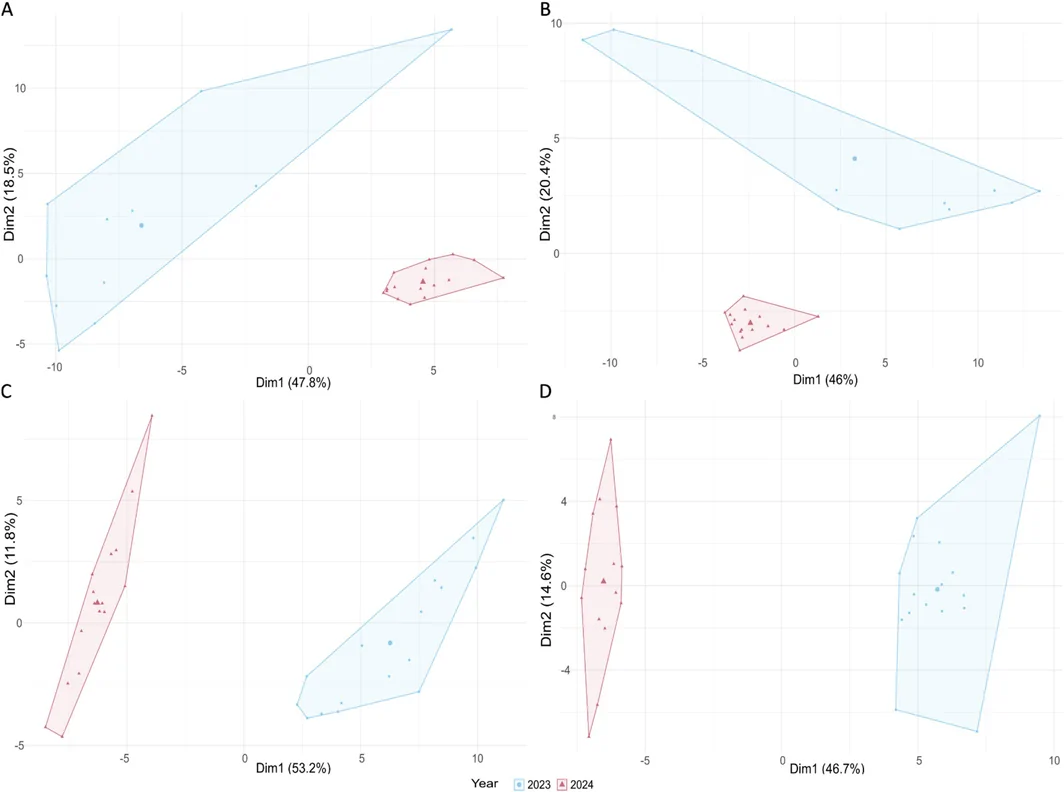
Principal Component Analysis (PCA) illustrating the year effects on wheat transcriptional profiles. PCA for the bread wheat cultivars (A) Venezio and (B) Soissons, and for the durum wheat cultivars (C) EPO122 and (D) EPO145.

### 3.2 Limited Impact of Cultivar Mixtures on Gene Expression Profiles in Wheat, With an Upregulation of Some Specific Genes in Durum Wheat Cultivars

In our study, we observed minimal effects of plant mixtures on gene expression profiles across all cultivars examined. The PCA plots revealed no separation pattern in transcriptional profiles between plants grown in mixtures versus pure conditions for either bread wheat or durum wheat cultivars (Figure S3). However, specific genes in durum wheat demonstrated significant differential expression when grown in mixtures. In the EPO122 line, the BSK gene, which is associated with brassinosteroid signaling, was downregulated under mixed conditions (Figure 3A). Similarly, in EPO145, two stress‐related genes—CHI4, a chitinase involved in plant defense, and DREB2B, a transcription factor linked to drought response—showed up‐ and downregulation, respectively, in mixture conditions (Figure 3B,C). In contrast, no differentially expressed genes were identified in the bread wheat varieties.

**FIGURE 3:**
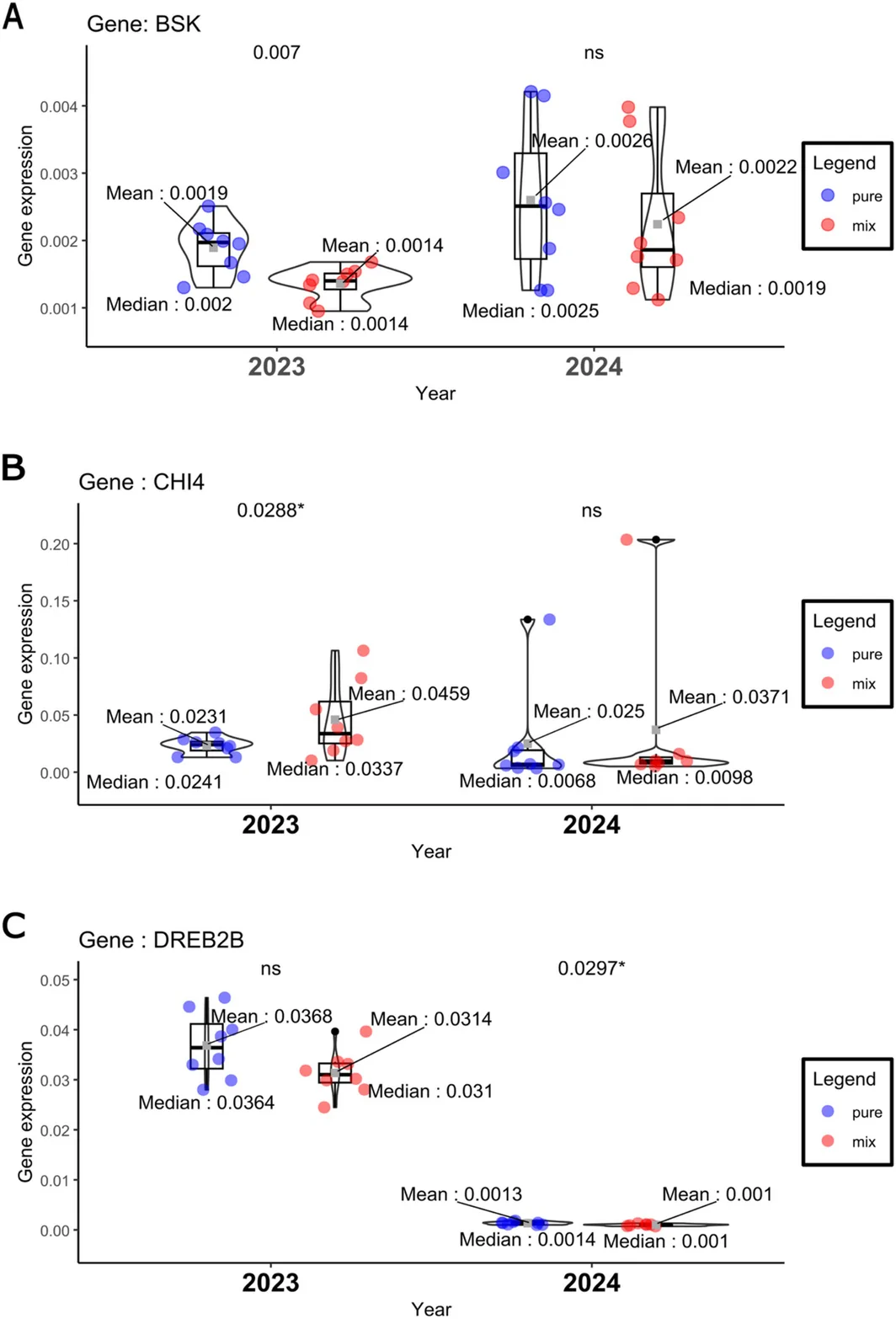
Boxplots of relative expression levels for genes significantly upregulated or downregulated in durum wheat cultivars grown under mixture conditions. (A) Expression of BSK in EPO122, associated with brassinosteroid signaling. (B) Expression of CHI4 in EPO145, related to defense mechanisms. (C) Expression of DREB2B in EPO145, a gene involved in drought response. Statistical analyses between pure and mixed conditions were conducted using non‐parametric ANOVA. Significance levels are indicated as follows: *p* < 0.1, *p < 0.05, **p < 0.01, ***p < 0.001.

### 3.3 Cultivar‐Specific Density‐Dependent Transcriptional Changes in Wheat

While no general density effect was observed in the transcriptional profiles of the durum wheat cultivars and the bread wheat Venezio (Figure S4), the analysis revealed a more pronounced effect of density on the transcriptional profiles of Soissons (Figure 4A). Nevertheless, all cultivars showed significant differential expression for specific genes in response to density variations (Figures S5 and S6).

**FIGURE 4:**
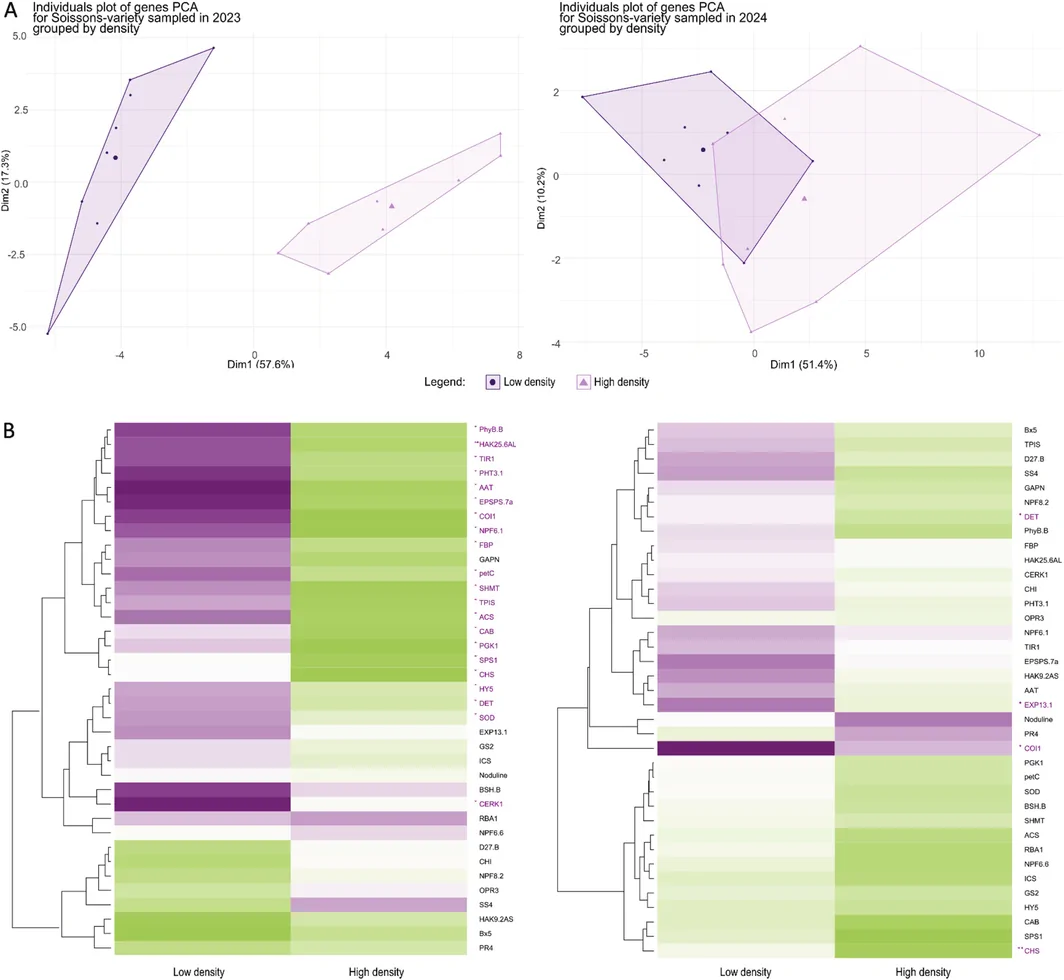
Transcriptional responses of the Soissons variety to density variations. (A) Principal Component Analysis (PCA) illustrating the density effect on the transcriptional profile of Soissons in 2023 (left panel) and 2024 (right panel). (B) Heatmaps of relative gene expression levels in Soissons grown under low or high‐density conditions in 2023 (left) and 2024 (right). Gene expression levels increase from green to white to purple. Statistical analysis was performed using a non‐parametric ANOVA followed by Benjamini‐Hochberg correction to test the density effect in each year. Genes showing a significant density effect are highlighted in purple. Significance levels are denoted as follows: *p* < 0.1, *p < 0.05, **p < 0.01, ***p < 0.001.

For EPO122, five differentially expressed genes were identified: three downregulated in high density (TPIS, petC, and NPF6.6) associated with carbon metabolism, photosynthesis, and nitrogen metabolism respectively, and two upregulated (FlaS and BSH.B) linked to flavonoid biosynthesis and shade avoidance syndrome (Figure S6B). EPO145 displayed three differentially expressed genes, with two downregulated (NPF6.1 and PCNA2) related to nitrogen metabolism and cell state, and one upregulated gene (SHMT) associated with carbon metabolism (Figure S6C). In Venezio, three upregulated genes were identified, two of which are involved in carbon metabolism (PGK1 and RBA1) and one in potassium metabolism (HAK7.2DS) (Figure S6A).

In Soissons, a total of 23 genes were downregulated under high density, primarily impacting primary metabolism (Figures 4B and S5). Notably, five out of eight carbon metabolism genes present on the chip were downregulated (TPIS, SHMT, PGK1, SPS1, and FBP). Additional downregulated genes included two associated with nitrogen metabolism (AAT and NPF6.1), three linked to photosynthesis (petC, PhyB‐B, and CAB), one related to potassium (HAK25.6AL), and one associated with phosphate (PHT3.1). Furthermore, two defense‐related genes (CERK1 and SOD) and two flavonoid‐related genes (CHS and EPSPS.7a) also showed downregulation, indicating a broader impact on both primary and, to a lesser extent, specialized metabolic pathways.

### 3.4 Interactions Between Density and Mixture Effects Impact Carbon Metabolism and Brassinosteroid Genes, With Different Expression Patterns Related to Wheat Cultivars

Our analysis revealed consistent effects of interactions between density and mixture on seven genes in 2023 and 2024 (Figure 5) in three out of the four cultivars examined: Venezio (Figure 5A), Soissons (Figure 5B), and EPO145 (Figure 5C). Among these seven genes, four were linked to carbon metabolism. These genes displayed two distinct expression patterns. First, differential expression depending on density, with SPS1 being downregulated at high density in Soissons, while TK was upregulated at high density in Venezio, where density effects were more pronounced in pure conditions for SPS1 and in mixed conditions for TK. Second, some genes demonstrated inversions in their expression patterns based on whether they were in pure or mixed conditions; for instance, PDC was upregulated under high density in mixed conditions but downregulated in pure condition for Venezio, and similarly, TK showed upregulation in mixed conditions but downregulation in pure conditions for EPO145. Moreover, the gene DET showed a significant density‐mixture interaction in both Soissons and EPO145, although the nature of this interaction differed between the two cultivars. The impact of mixtures on wheat physiology therefore depends on density and variety.

**FIGURE 5:**
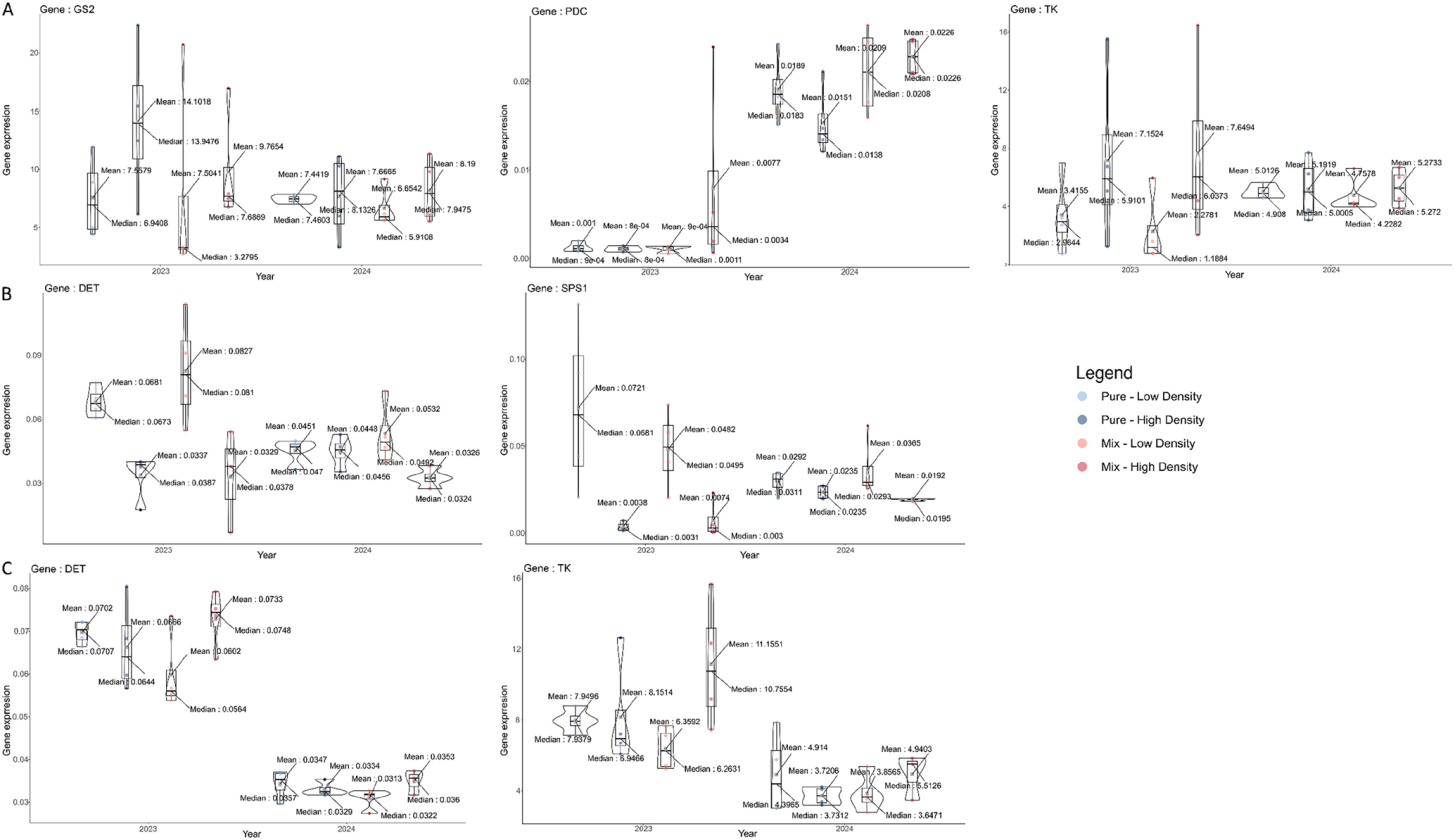
Boxplots of relative expression levels for genes with density and mixture interactions across three cultivars: (A) Venezio, (B) Soissons, and (C) EPO145. The boxplots illustrate the relative expression levels of genes exhibiting consistent interaction trends between density and mixtures across the two years.

## 4 Discussion

In this study, we evaluated whether molecular responses can be reliably captured under semi‐controlled conditions. We examined how sowing density and cultivar mixtures can influence the transcriptional profiles of both durum and bread wheat. Conducted over two years under semi‐controlled mesocosm conditions, this experiment aimed to simulate field‐like environments while maintaining precise control over sowing designs and ensuring genotype identification. Our results revealed significant annual variability, underscoring the complexity of gene expression responses under changing environmental conditions. Despite this, we identified subsets of genes with consistent expression trends across both years, showing effects of sowing density and density‐mixture interactions on gene expression. However, the direct impact of mixtures on studied cultivars' transcriptional profiles appeared to be relatively limited.

### 4.1 Transcriptional Profiles Are Affected by Year‐Specific Environmental Conditions

Performing molecular analyses in field‐like conditions introduces a multitude of variables that can obscure the interpretation of results. Environmental factors such as temperature, precipitation, humidity, sunlight, and microclimate variations all contribute to the modulation of plant transcriptomes (Hashida et al. 2022), making it challenging to isolate specific effects. For instance, our meteorological data revealed that the range between minimal and maximal temperatures was broader in 2023 compared to 2024, with colder temperatures observed in 2023. Cold stress is known to impact the wheat transcriptome (Zhao et al. 2019), suggesting that the cooler conditions in 2023 may have contributed to the higher dispersion of wheat transcriptomes in that year and to the distinct transcriptional profiles observed between the two years. This annual variability highlights the prominent role of environmental fluctuations in shaping gene expression patterns, complicating the detection of smaller, more subtle effects, such as those associated with cultivar mixtures at the seedling stage.

### 4.2 Limited Gene Expression Response to Mixtures, Potentially Masked by External Environmental Factors

In this experiment, the mixture effects on gene expression were relatively limited, as only three genes showed significant differential expression in response when another genotype was the neighbor. Notably, one gene involved in brassinosteroid signaling and two genes related to stress responses were differentially regulated under the mixture treatment. Given that mixture effects on transcriptional profiles were observed under controlled conditions (Pélissier et al. 2023), the limited number of genes responding in this semi‐controlled environment suggests a potentially narrow regulatory response, which could be overshadowed by stronger environmental factors.

### 4.3 Density Impacts Wheat Transcriptional Responses but in Variable Ways

Our findings revealed that plant density had an impact on carbon metabolism, with notable variation in responses observed among the different plant cultivars. Specifically, in Soissons, high sowing density led to a downregulation of primary metabolism. This contrasts with previous molecular studies, which mainly reported an upregulation of primary metabolism under high‐density conditions (Geisler et al. 2012; Masclaux et al. 2012; Dai et al. 2014). However, our findings are consistent with morphological studies that have shown reductions in both above‐ and below‐ground growth, as well as total biomass, at increased densities (Muñoz‐Parra et al. 2017; Postma et al. 2021). Moreover, the downregulation of primary metabolism observed in Soissons is further supported by the documented decrease in photosynthetic rates under high‐density conditions (Postma et al. 2021). Based on these results, we can speculate that Soissons is not a competitive variety, but is instead adapted to perform well without strong competitive responses at high plant densities—a trait that may be desirable in modern wheat cultivars selected for dense cropping systems. In addition, these observations underscore the cultivar‐specific nature of plant responses to density.

### 4.4 Interactions Between Density and Mixture Are Complex

The interaction between sowing density and mixture condition influenced the expression of genes related to brassinosteroid signaling and carbon metabolism. Brassinosteroids are key plant hormones that regulate shade‐avoidance responses, the best‐characterized mechanism by which plants perceive and respond to neighboring vegetation (Huber et al. 2021). This response typically promotes stem elongation and other architectural changes, which can in turn alter carbon allocation and overall metabolic activity. Our findings indicate that the effects of sowing density on these pathways are not uniform; they vary depending on whether crops are grown in pure or mixed stands. Moreover, these density‐mixture interactions are gene‐specific and vary across different genotypes. Indeed, gene expression responses can be amplified, diminished, or even reversed depending on the specific combination of sowing density and mixture composition. This underscores the importance of considering multiple environmental factors, such as sowing density and mixtures alongside several cultivars when studying plant responses. Furthermore, the complexity of these interactions emphasizes the need for advanced omics tools used in kinetics, including time‐resolved transcriptomics, proteomics, or metabolomics, to elucidate the interplay between density, mixture composition, and variety.

## 5 Conclusion

This study highlights the intricate dependencies of plant responses on genetic and environmental factors, specifically sowing density, cultivar mixtures, and their interactions. While the observed effects were often modest, they may have been masked by stronger environmental effects. This study confirms that expression analysis experiments conducted under semi‐controlled conditions are not readily reproducible across one year and calls for caution when conducting this type of analysis. This study also shows that expression profiles are highly dependent on the genotypes tested, limiting the generalization of these results. Future studies would benefit from integrating mesocosm transcriptional data with agronomic performance traits to better connect molecular variation with functional outcomes.

## Funding

This work was supported by the French National Research Agency under the Investments for the Future Program [Grants MOBIDIV project ANR‐20‐PCPA‐0006].

## Conflicts of Interest

The authors declare no conflicts of interest.

## Supporting information


**Figure S1:** Precipitations, and minimum, maximum, and average temperatures during the two growing seasons: (a) 2023 and (b) 2024. Meteorological data was obtained through an INRAE meteorological station located approximately 800 m away from the experimental site.

**Figure S2:** Overview of biological processes represented in the RT‐qPCR chip for wheat transcriptional response analysis. The RT‐qPCR chip is designed to assess the transcriptional responses in wheat by targeting a comprehensive selection of genes involved in multiple biological processes. The chip includes genes associated with defense (15 genes), carbon metabolism (10), nitrogen metabolism (9), cell state (8), housekeeping functions (5), flavonoid biosynthesis (5), cytokinin signaling (4), photosynthesis (3), phosphate metabolism (3), potassium regulation (3), jasmonic acid signaling (3), gibberellic acid signaling (3), ethylene signaling (3), drought response (3), DIMBOA biosynthesis (3), brassinosteroid signaling (3), auxin signaling (3), abscisic acid signaling (3), strigolactones (2), shade avoidance syndrome (2), and salicylic acid signaling (2). The full list of genes is detailed in Table S1.

**Figure S3:** Principal Component Analysis (PCA) showing the mixture effects on wheat transcriptional profiles. PCA for two bread wheat cultivars (a) Venezio and (b) Soissons, and two durum wheat cultivars (c) EPO122 and (d) EPO145 in 2023 (left panel) and 2024 (right panel).

**Figure S4:** Principal Component Analysis (PCA) showing the density effects on wheat transcriptional profiles. PCA for the bread wheat cultivars (a) Venezio, and two durum wheat cultivars (b) EPO122 and (c) EPO145 in 2023 (left panel) and 2024 (right panel).

**Figure S5:** Transcriptional responses of the Soissons variety to density variations. Boxplots of relative expression levels for genes significantly downregulated in Soissons grown under high density conditions. Statistical analyses between high‐ and low‐density conditions were conducted using non‐parametric ANOVA. Significance levels are indicated as follows: *p* < 0.1, *p < 0.05, **p < 0.01, ***p < 0.001.

**Figure S6:** Boxplots of differentially expressed genes in response to density effects for (a) Venezio, (b) EPO122, and (c) EPO145. Statistical analyses between high‐ and low‐density conditions were conducted using non‐parametric ANOVA. Significance levels are indicated as follows: *p* < 0.1, *p < 0.05, **p < 0.01, ***p < 0.001.

**Table S1:** Detailed list of genes included in the RT‐qPCR chip for analyzing transcriptional responses in wheat ( *Triticum aestivum* and *Triticum turgidum* ). This chip is designed to capture a broad spectrum of biological processes by incorporating genes involved in defense mechanisms, carbon and nitrogen metabolism, cell state regulation, housekeeping functions, phytohormone signaling (cytokinin, jasmonic acid, gibberellic acid, ethylene, abscisic acid, brassinosteroids, auxin, and strigolactones), as well as processes such as flavonoid biosynthesis, phosphate and potassium metabolism, photosynthesis, drought response, DIMBOA biosynthesis, and shade avoidance syndrome. For each gene, the table provides the gene name, full description, associated biological process, specific sub‐process, gene IDs in both *T. aestivum* and *T. turgidum* , reference publication, and the expected transcriptional response within the analyzed processes.

**Table S2:** List of genes showing consistent expression patterns across years for each wheat cultivar (Venezio, Soissons, EPO122, and EPO145) and for each effect (density or mixture).

**Table S3:** Normalized gene expression data for each wheat cultivar (Venezio, Soissons, EPO122, and EPO145) under the density and mixture treatments in 2023 and 2024. Expression values were normalized using the two housekeeping genes Tubulin and Ta54227.

## Data Availability

The data that supports the findings of this study are available in Table S3.
